# IMPROVING HEALTHCARE REGULATIONS FOR RESIDENTS WITH DEMENTIA: PERSPECTIVES OF REGISTERED DIETITIANS

**DOI:** 10.1093/geroni/igad104.3425

**Published:** 2023-12-21

**Authors:** Phatt Thaitrong, Joy Douglas, Linda L Knol, Amy Ellis, Hyunjin Noh, Shena Sanchez

**Affiliations:** The University of Alabama, Tuscaloosa, Alabama, United States; The University of Alabama, Tuscaloosa, Alabama, United States; The University of Alabama, Tuscaloosa, Alabama, United States; The University of Alabama, Tuscaloosa, Alabama, United States; The University of Alabama, Tuscaloosa, Alabama, United States; The University of Alabama, Tuscaloosa, Alabama, United States

## Abstract

In order to admit residents on Medicare or Medicaid, long-term care (LTC) facilities in the United States are required to comply with federal regulations provided by the Centers for Medicare and Medicaid Services (CMS). However, limited research exists regarding the first-hand experiences of Registered Dietitians (RDs) working in LTCs and their perceptions on the extent to which the current healthcare regulations adequately address the nutritional needs of residents with dementia. Exploring the perspectives of RDs is crucial since residents with dementia may face difficulties expressing their nutritional needs. Our study explored RDs’ perspectives and identified how menu planning (F803) and mealtime environment (F558) regulations could be improved to better meet the needs of LTC residents with dementia. RDs who work with residents with dementia in the U.S. (n = 18) participated in one of five focus groups. Focus groups were led by a trained moderator using a semi-structured interview guide, transcribed verbatim, and audited by the research team for accuracy. Transcripts underwent descriptive content analyses using NVivo© software. Analyses revealed the RDs’ suggestions that F803 and F558 regulations need to 1) be more clear, 2) specify objective criteria to measure adherence, 3) work toward patient-centered care, and 4) give priority to residents with dementia. To meet the needs of LTC residents with dementia, mealtime-related healthcare regulations need to incorporate clear verbiage with measurable criteria. This will allow for easier understanding of the regulations and assessment of compliance with the regulations in LTCs.

